# Robot-Assisted Repair of Bladder Rupture following Penile Ring Entrapment

**DOI:** 10.1155/2023/5523569

**Published:** 2023-09-07

**Authors:** Matthew Skalak, Rami Jirjis, Barrett G. Anderson, Brandi D. Miller

**Affiliations:** Detroit Medical Center Urology Residency, Harper Professional Building, 4160 John R St., Suite 1017, Detroit, MI 48201, USA

## Abstract

Penile rings have been used to help sustain erection and enhance sexual pleasure for centuries. Constriction of the penis reduces the outflow of blood from the cavernosal tissue. However, if left for an extended time period, a condition called penile ring entrapment can occur. This may result in severe edema, gangrene, necrosis, and even penile amputation. Penile ring entrapment is a very rare condition; complete urinary obstruction with concomitant bladder rupture as a result renders this case even more extraordinary. We discuss our experience in the management of a 64-year-old man, who presented with altered mental status and inability to urinate, found to have penile ring entrapment and intraperitoneal bladder rupture. Removal of the constricting ring was performed in the ED, and bladder injury and penile necrosis were subsequently repaired with robot-assisted laparoscopic cystorrhaphy, penectomy, and perineal urethrostomy.

## 1. Background/Introduction

Penile rings have been used for the purpose of enhancing sexual pleasure for centuries. They function by decreasing the outflow of blood from the erectile tissue, which results in sustained erection. Various materials such as wedding rings, rubber bands, “bull rings,” and plastic bottlenecks have been used [1, 2]. Penile rings should not be applied longer than 30 minutes [3, 4], as prolonged constriction can cause penile ring entrapment. This condition leads to severe penile ischemia, edema, and tissue necrosis. In such cases, urgent medical intervention is necessary to remove the offending ring to restore penile perfusion. In the emergency setting, various items have been used to remove penile rings including lubricants, diamond-tip Midas surgical drills, stout scissors, K-wire cutters, Channellock EMT/firefighter cutters, bone cutters, and even nonsurgical drills [5].

The present case is particularly unusual given the initial presentation of concomitant bladder injury. Injury to the bladder is most commonly associated with iatrogenic injuries during pelvic or gynecologic procedures, as well as blunt and penetrating trauma [6]. Management of bladder rupture often depends on whether the defect results in extra- versus intraperitoneal urine leakage; respectively, treatment can range from simple Foley catheter drainage to open surgical repair [7].

## 2. Case

**Figure 1:**
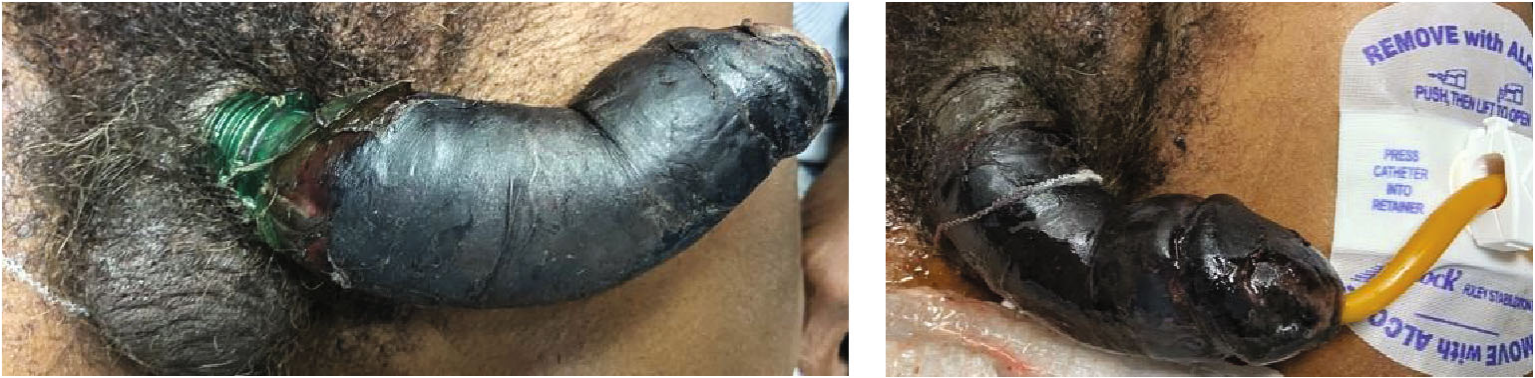
Initial presentation. On initial examination, a soda bottle found at the penile base was causing strangulation. The penis appeared entirely necrotic. Meatal stenosis was noted. The bottleneck was removed from the patient's penile shaft with cardiac wire cutters, and a Foley catheter was placed over a wire following urethral dilation.

A 64-year-old male with a history of schizophrenia presented to the emergency department due to altered mental status and reported inability to urinate for 48 to 72 hours due to penile ring entrapment. Further questioning revealed the patient had attempted to masturbate using a 16-ounce soda bottle, which resulted in penile edema and subsequent entrapment. Initial physical examination revealed meatal stenosis, a bottleneck constricting the proximal penile shaft with findings concerning for penile dry gangrene (Figure 1). The bottleneck was promptly and carefully removed atraumatically using a cardiac wire cutter.

**Figure 2:**
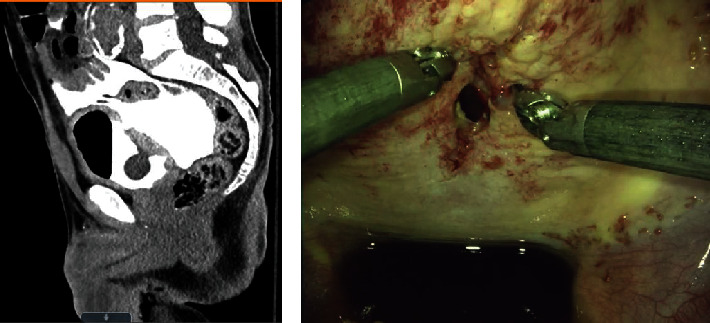
Intraperitoneal bladder injury. Computed tomography cystogram sagittal view demonstrated an intraperitoneal bladder injury at the dome. Direct robot-assisted laparoscopic visualization is shown. Repair was performed in a standard 2-layer fashion using 3-0 and 2-0 V-Loc™ sutures.

A basic metabolic panel revealed an elevated serum creatinine of 11 mg/dL. Bladder drainage was established with a Foley catheter after gentle dilation of associated meatal stenosis with an initial output of 300 mL of clear, light pink urine. The abdominal examination at the time of catheter placement was soft, nontender, and without signs of peritonitis. However, a bedside abdominal ultrasound performed by the emergency department physicians demonstrated free peritoneal fluid. A CT cystogram demonstrated contrast surrounding small bowel loops and a discontinuity of the bladder dome consistent with an intraperitoneal bladder rupture (Figure 2(a)).

Given the radiographic findings, surgical repair was recommended. Due to the isolated and straightforward nature of the defect, we proceeded with prompt surgical repair of the bladder injury with a minimally invasive approach using a DaVinci Robot™ (Intuitive Surgical, Sunnyvale, CA). Insufflation of the peritoneal cavity to 15 mmHg was established with a Veress needle, and a supraumbilical robotic trocar was placed to serve as the camera port. Three additional 8 mm robotic trocars—one on the left and two on the right—were placed in line with the initial trocar, spaced evenly in a manner like robot-assisted radical prostatectomy. A final 12 mm laparoscopic trocar was placed in the left lower quadrant as an assistant port. After docking, copious intraperitoneal urine was evacuated using a laparoscopic suction, and a roughly 3 cm defect was readily identified at the bladder dome (Figure 2(b)). The bladder defect was repaired in a standard running 2-layer fashion using 3-0 and 2-0 V-Loc™ (Medtronic, Minneapolis, MN) sutures. The repair was confirmed to be watertight.

**Figure 3:**
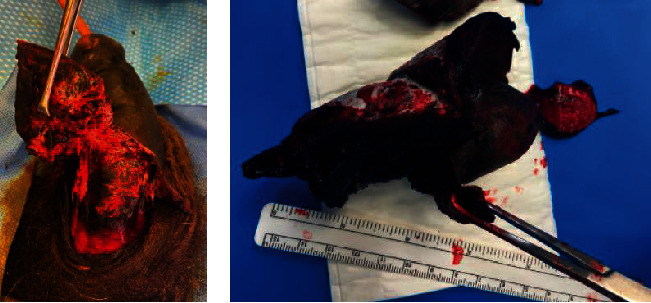
Initial debridement of the penile shaft. Intraoperative and back table dissection demonstrated dry necrosis throughout most of the penile shaft and glans.

**Figure 4:**
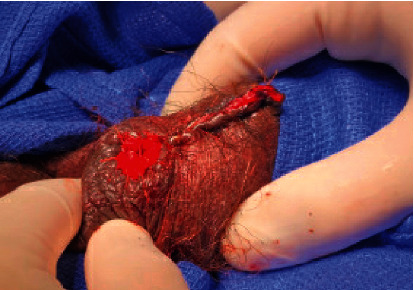
Partial penile amputation. Performed on hospital day 2 with creation of a 1.5 cm penile stump.

**Figure 5:**
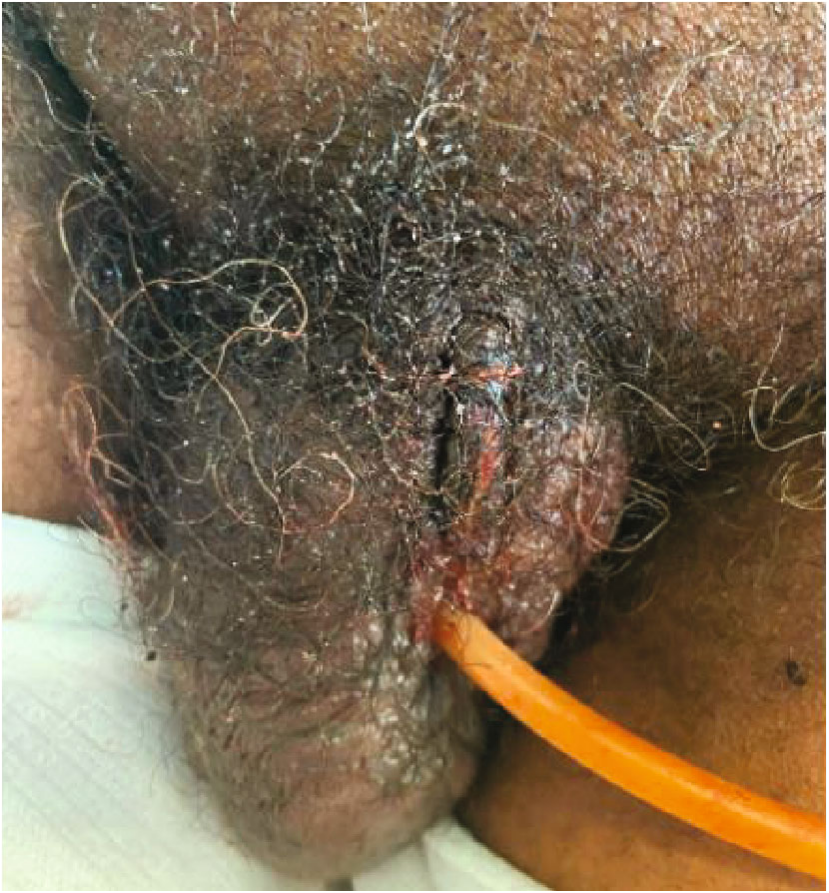
Post-partial penile amputation. As seen on postoperative day 7 following partial penile amputation.

After a period of observation, the patient was brought back to the operating room 48 hours later for penile debridement. Figures 3 and 4 demonstrate dry necrosis of most of the patient's penile shaft and glans. Due to the extensive necrosis of the penile tissue, the phallus was not salvageable and required a partial penile amputation leaving a 1.5-inch penile stump to preserve the ability to urinate standing up. The corporal bodies were closed with horizontal mattress stitches of 2-0 Vicryl™ (Ethicon Inc., Bridgewater, NJ). The overlying penoscrotal skin was debrided to healthy tissue and closed longitudinally using horizontal mattress stitches of 3-0 Chromic Gut suture (Ethicon Inc., Bridgewater, NJ). The proximal urethra was spatulated in the usual fashion and matured to the skin using interrupted 4-0 Vicryl™ suture. The patient was observed closely during his hospitalization, and culture-appropriate antibiotics were continued through discharge per recommendations from the infectious disease physicians.  Figure 5 notes the healing process on postoperative day 7.

**Figure 6:**
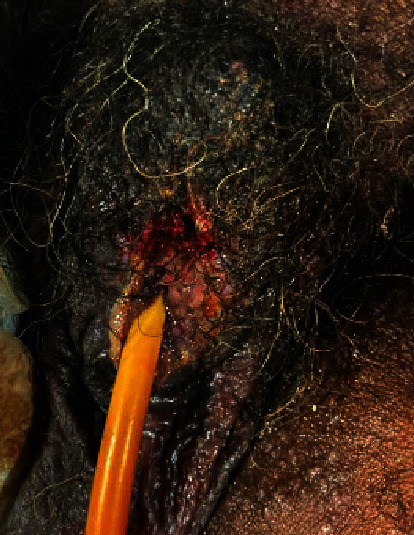
Penile stump necrosis. On hospital day 14, postoperative day 13 from index operation, necrotic changes were noted to the penile stump.

**Figure 7:**
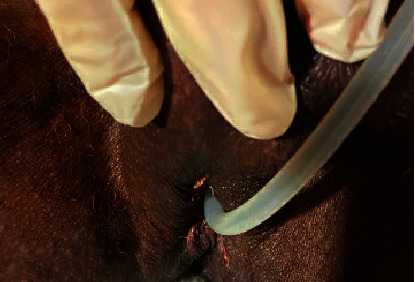
Healing perineal urethrostomy. Urethrostomy able to accommodate a 30F Bougie.

Due to comorbid medical and psychiatric conditions, the patient remained inpatient until hospital day 14, 13 days after the index operation. On this day, new necrosis of the urethra was discovered, as pictured in Figure 6. The patient had intermittent mild tachycardia but otherwise did not report or exhibit any symptoms or laboratory findings concerning for a progressing infectious process. The patient was taken back to the operating room for excision of the necrotic urethra and a perineal urethrostomy on hospital day 16. Necrosis of the bilateral corporal bodies, corpus spongiosum, and urethra was noted down to the level of the bulbar urethra, with involvement of the anterior scrotum as well. The perineal urethrostomy easily accommodated a 30 French bougie, and the external sphincter was intact. The urethrostomy healed well (Figure 7). However, the patient developed a small, localized abscess under the anterior scrotal suture line, which required incision and drainage 20 days after the index operation, which was then managed with daily packing changes. The patient was ultimately discharged to a skilled nursing facility.

## 3. Discussion

Penile rings restrict the outflow of blood from the penile tissue resulting in a sustained erection for sexual pleasure. Improper use, however, can lead to tissue ischemia, edema, necrosis, tissue loss, and even death [8]. Penile ring entrapment resulting in penile loss and bladder rupture is rare. Most patients present within a reasonable timeframe to allow for restoration of blood flow to salvage penile tissue. Delayed presentation for medical treatment may be the result of embarrassment or, in the case of our patient, comorbid psychiatric conditions. Unfortunately, our patient's marked delay in presentation contributed to catastrophic tissue decline; the prompt removal of the plastic bottleneck upon presentation could not reverse tissue necrosis and the need for eventual penile amputation.

The uniqueness of this case involves not only the sheer extent of penile damage necessitating penile amputation but also the associated bladder rupture. Bladder injuries are more common than penile ring entrapments; however, they are more likely to be associated with trauma or iatrogenic injury. Clinical suspicion was raised in the present case due to a severely elevated serum creatinine level in the setting of prolonged urinary retention with only 300 mL of initial urine output upon Foley catheter placement.

Management of bladder rupture has been thoroughly described by the American Urological Association [7]. In our case, the CT cystogram clearly indicated an intraperitoneal rupture at the bladder dome. Our options were to perform either an open or minimally invasive bladder repair, as management by Foley catheter drainage alone would have likely been insufficient for bladder healing. We ultimately pursued robot-assisted laparoscopic repair given the isolated and relatively straightforward nature of the bladder defect and clinical presentation. We feel that this approach can facilitate patient recovery by minimizing blood loss and decreasing post-operative pain and length of stay [9]. Despite these potential benefits, the lengthy duration of our patient's hospitalization was more related to the penile injury than the bladder rupture; progressive penile necrosis led to the need for removal of most of the patient's penile tissue followed by a perineal urethrostomy.

While rare, urologists should be aware of the possibility of bladder rupture in the setting of prolonged penile ring entrapment. When possible, we feel that a minimally invasive approach, whether laparoscopic or with robot assistance, is appropriate when the rupture is straightforward and lends itself well to such a technique. With respect to penile necrosis, preservation of tissue should be the goal to maintain urinary and sexual function; when necessary, however, a perineal urethrostomy may be performed if the necrosis is extensive.

## Data Availability

Additional data regarding this case is not publicly available in order to protect patient anonymity.
